# Exploring resilience as a moderator of social media appearance activity and body image concerns in adolescents

**DOI:** 10.1038/s41598-026-45442-z

**Published:** 2026-04-09

**Authors:** Nikol Kvardova, Anna Literova, Hana Machackova

**Affiliations:** Interdisciplinary Research Team on Internet and Society, Faculty of Social Studies, Masaryk University, Jostova 10, Brno, Czech Republic

**Keywords:** Appearance activity on social media, Body image, Resilience, Media ideals, Negative appearance feedback, Adolescents, Neuroscience, Psychology, Psychology

## Abstract

Considering their developmental sensitivities to peer approval and heightened self-consciousness, many adolescents experience body image concerns when engaging with visual social media. Using a sample of 885 adolescents aged 15–19 (*M* = 16.99, *SD* = 1.19; 61% girls, 39% boys), this study explored whether resilience to idealized media bodies and to negative appearance feedback buffered the links between social media appearance activity, body esteem, and self-objectification. While resilience to negative appearance feedback did not significantly moderate these associations, resilience to media ideals did, but in an unexpected direction. Adolescents with higher-than-average resilience who engaged more frequently in appearance activity reported greater self-objectification and lower body esteem, whereas those with lower-than-average resilience reported less self-objectification and higher body esteem. These results emerged for both girls and boys. Even though these results should be taken as preliminary, they suggest that resilience may play a more intricate role in adolescents’ social media appearance activity and body image. Future studies should investigate this notion further and also acknowledge the reverse path, where resilience may build up from exposure to idealized bodies on social media.

## Introduction

Adolescents regularly engage in so-called *appearance activity* on visual social media platforms (e.g., Instagram, TikTok). This activity encompasses commenting on and posting images of idealized bodies (e.g., fitness-, eating-, and beauty-focused posts), whether their own or browsing such content posted by their friends^1^. Although late adolescents appear less vulnerable to body image concerns than younger ones^2^, they remain frequently exposed to visual social media, which continues to play a prominent role in shaping how they perceive their bodies^3^. Given that these platforms predominantly present selectively posted and unrealistically attractive bodies, appearance activity has been found to worsen body image in adolescents^4^. This applies to both browsing the flawless appearances of others and to creating and sharing idealized self-presentations^5^.

The present study examines whether resilience to body ideals in media and to negative appearance feedback serve as factors that buffer the associations between appearance activity on social media and body image concerns, that is, heightened self-objectification and decreased body esteem, in adolescents. This investigation responds to the voiced need to identify the factors that protect against body image concerns associated with engagement with visual social media^6^. Resilience to body ideals in media and to negative appearance feedback has been highlighted as key to adolescent positive body image^7^, but it has been scarcely studied. These results may preliminarily inform future intervention and research efforts by showcasing whether such resilience protects adolescents from risks associated with visual social media engagement.

Whereas *body esteem* refers to the self-evaluation of physical appearance^8^, *self-objectification* encompasses the perception of the body from a third-person perspective and valuing oneself predominantly based on physical attractiveness^9^. Both are common during adolescence and are connected to deteriorated health outcomes. Decreased body esteem (in the literature predominantly labelled as body dissatisfaction) and heightened self-objectification have been linked with depressive^10,11^ and anxiety symptoms, lower self-esteem^12^, body shame^11^, dieting, unhealthy weight control behaviors, binge eating^13^, and even the onset of eating disorders^14^. These body image concerns can be significantly exacerbated by visual social media, which often emphasize attractiveness as highly important and promote unattainable appearance ideals^15^. Previous research has shown that various appearance activities on social media are associated with decreased body esteem, like engaging with photos on Facebook^16^, editing and browsing selfies^17,18^, using appearance-based platforms like Instagram and Snapchat^4,19^, and liking and commenting on others’ updates and photos on social media^20^. Furthermore, viewing others’ selfies and editing and investing in one’s own selfies were associated with more self-objectification in adolescents^18,21^. While posting one’s selfies and other content on social media has so far revealed less support for worsening body esteem and heightening self-objectification^18,20,21^, it still contributes to body image concerns^22^.

Appearance activity on social media has been linked to poorer body image in adolescents^19^, but the factors that may buffer this link have been scarcely studied. One such salient buffer may be resilience to body ideals in the media and resilience to negative appearance feedback, defined by employing coping strategies, such as distracting oneself or shifting attention away from these ideals and negative comments received about one’s body (e.g., teasing, peer criticism) and dismissing them as unimportant^7^. Adolescents have reported the importance of adopting these strategies in protecting their body image from social media, like disregarding negative comments on their appearance^23^, actively avoiding, critically evaluating, and positively reframing idealized content, and selecting positive content^24–26^. Although social media literacy—characterized by the critical evaluation of social media content—has been preliminarily suggested to protect body image from the effects of idealized images^27^, specific strategies that adolescents employ when they encounter such content may be more important^26^. These strategies may especially empower adolescents to protect themselves from body image concerns when engaging with appearance-related content on social media that promotes unattainable ideals and may also expose them to negative comments. However, research on these strategies that constitute resilience to media ideals and negative appearance-related feedback in the context of engagement with appearance-focused social media content remains limited.

Previous literature has indicated that the overall impact of social media on body image seems similar among adolescent girls and boys^28^, although girls typically face more appearance pressures and objectification as they grow up and when they use visual social media^15^. Compared to boys, girls also report employing fewer protective strategies when on social media^26^ and taking appearance-related content, such as comments, more seriously for their appraisals of attractiveness and resultant body image^29^. On the other hand, resilience can build up from challenging experiences^30,31^, such as receiving criticism and facing media ideals. Girls, who experience these more often^15^, may be more empowered to shield themselves from appearance activity on social media. Given the limited research on *gender differences* in the buffering role of resilience between social media appearance activity and body image concerns, this study aimed to address this gap.

Overall, three research questions were formulated:

- *RQ1:* Does resilience to media ideals buffer the relationship between appearance social media activity and body image concerns (i.e., lower body esteem, higher self-objectification) in adolescents?
- *RQ2:* Does resilience to negative appearance feedback buffer the relationship between social media appearance activity and body image concerns (i.e., lower body esteem, higher self-objectification) in adolescents?
- *RQ3:* Are there differences between girls and boys in the buffering role of resilience to media ideals and to negative appearance feedback between social media appearance activity and body image concerns (i.e., lower body esteem, higher self-objectification)?

## Methods

### Participants and procedure

The sample consisted of 885 adolescents aged 15–19 (*M* = 16.99, *SD* = 1.19; 61% girls, 39% boys). This study was approved by the Research Ethics Committee of Masaryk University (ref. number EKV-2022-098) and conducted in line with the principles of the Declaration of Helsinki. The data were collected in nine high and vocational schools and seven grammar schools in the South Moravia region of Czechia between November and December 2022. Trained research assistants administered the data collection via online questionnaires in classrooms within a dedicated 45-min session. All adolescents aged 18 and above provided their own written Informed consent, while those under 18 participated with written Informed consent from a parent or legal guardian. Additionally, all participants provided oral informed consent before filling out the questionnaire.

The first page of the questionnaire described the study’s purpose and contents, emphasizing that responses were anonymous, participation was voluntary, and participants could withdraw at any time. Adolescents were assured of anonymity. They could skip questions and respond with *I don’t know* or *Prefer not to say*. The questionnaire covered topics such as demographics, body image, social media use, body-related comments from friends and family, self-esteem, self-concept clarity, and attachment scales. The mean completion time was 25 min. Items within each scale were presented in a randomized order. The questionnaire items were collaboratively translated into Czech by the authors and an additional researcher involved in data collection. The first author and the additional researcher drafted multiple translation versions. The final translations were selected in collaboration with the other authors. The study was preregistered as an exploratory study (see https://osf.io/5qzja/overview?view_only=e37ff85fbaa64bffa5f65d24e4aaea33).

### Measures

#### Appearance activity on social media

Appearance activity on social media was measured with the Social Media Appearance Preoccupation Scale: Appearance Activity Subscale^1^. The scale included six items: three asked about the adolescents’ active engagement with appearance content (e.g., *When on social media I post, comment on, share, or like content about what and when to eat*); and three asked about browsing appearance content shared by their friends (e.g., *When on social media my friends post, comment on, share, or like content about what they would like their bodies to look like*). Answers ranged from (1) Strongly disagree to (7) Strongly agree. A higher score indicated higher appearance activity on social media (*M* = 2.80, *SD* = 1.56). The scale showed adequate internal consistency (McDonald’s ω_t_ = 0.82) and gender measurement invariance (see Results). The original validation study supported the expected one-dimensional factor structure, concurrent validity (as evidenced by associations with appearance anxiety and disordered eating), incremental validity, and gender measurement invariance^1^.

#### Body esteem

Body esteem was measured with the Body-Esteem Scale for Adolescents and Adults^8^. Ten items asked adolescents about their body esteem (e.g., *I like what I look like in pictures*) from 1 (Never) to 5 (Always). A higher score indicated higher body esteem (*M* = 2.86, *SD* = 0.47). The scale demonstrated strong internal consistency (McDonald’s ω_t_ = 0.91) and gender measurement invariance. Previous validation studies reported expected one-dimensional factor structure and concurrent validity (as evidenced by links with BMI, body appreciation, self-esteem, and disordered eating symptoms) in adolescent samples^32,33^. However, they also identified certain concerns, particularly content-overlapping and residually correlated item pairs^33^.

#### Self-objectification

Self-objectification was measured with the Self-Objectification Beliefs and Behaviors Scale^9^, which has two dimensions: the observer’s perspective on the body (seven items; e.g., *When I look in the mirror, I notice areas of my appearance that I think others will view critically*); and thinking of the body as representing the self (seven items; e.g., *How I look is more important to me than how I think or feel*). The answer scale ranged from 1 (Strongly disagree) to 5 (Strongly agree). The two dimensions were combined into a higher-order self-objectification factor for use in the main analysis. A higher score indicated higher self-objectification (*M* = 2.67, *SD* = 0.84). The scale showed adequate internal consistency (McDonald’s ω_t_ = 0.88) and gender measurement invariance. Although evidence from previous validation studies in adolescent samples is lacking, research conducted with young adults has reported high internal consistency and multiple aspects of validity, including two-factor structure, concurrent validity with appearance-contingent self-worth, appearance orientation, and internalization of sociocultural appearance standards^9,34^, and gender measurement invariance^34^.

#### Resilience to body ideals in media

Resilience to body ideals in media was measured with a subscale of the Positive Body Image in Adolescents Scale^7^. Two items (e.g., *If I am confronted with body ideals (e.g., slim or muscular bodies) in the media, I (would) try to distract myself with other things I like about the media*), assessing resilience to idealized images in general media, were answered on a scale from 1 (Strongly disagree) to 7 (Strongly agree). A higher score indicated higher resilience (*M* = 2.90, *SD* = 1.95). The scale demonstrated sufficient internal consistency (McDonald’s ω_t_ = 0.90).

#### Resilience to negative appearance feedback

Resilience to negative appearance feedback was measured with a subscale of the Positive Body Image in Adolescents Scale^7^. Four items (e.g., *If I (would) receive negative feedback on my appearance (e.g., from friends), it is important that I forget about it*) were answered on a scale that ranged from 1 (Strongly disagree) to 7 (Strongly agree). A higher score indicated higher resilience (*M* = 4.32, *SD* = 1.66). The scale demonstrated sufficient internal consistency (McDonald’s ω_t_ = 0.78) and gender measurement invariance. Additional evidence for factor structures and concurrent validity with body image variables has been reported in adolescent samples^7,35^.

#### Gender

Gender was determined with "You are:" with possible answers “A girl” (59%), “A boy” (38%), or “Other” (3%).

#### Age

Age was assessed with the question "How old are you?" followed by an open-response field for adolescents to enter their age.

### Data analysis

All analyses were conducted in *R* (version 4.5.0)^36^, using the packages *lavaan*^37^, *semTools*^38^, and *psych*^39^. The scales’ internal consistencies, descriptive statistics, zero-order correlations, and measurement invariance between girls and boys were examined. For measurement invariance, configural (i.e., fixed factor structure), metric (i.e., fixed factor loadings), and scalar (i.e., fixed intercepts) levels were compared using Root Mean Square of Approximation (RMSEA), Standardized Root Mean Square Residual (SRMR), the Comparative Fit Index (CFI), and the Tucker-Lewis Index (TLI). Rather than rely solely on conventional fit index thresholds^40,41^, we also assessed the model fit with Dynamic Measurement Invariance (DMI) cutoffs (using shiny application, see https://www.dynamicfit.app/MI/), which customize fit index difference thresholds (i.e., ΔCFI, ΔRMSEA, ΔSRMR) for the specific model characteristics and allows for more accurate and context-sensitive decisions about invariance^42^. The standard and DMI cutoffs are listed in Appendix A.

Using Structural Equation Modeling (SEM), a multi-group model with gender (i.e., girls and boys) as a grouping variable and Robust Maximum Likelihood estimation (MLR) was used. The missing data were handled using Full Information Maximum Likelihood (FIML), which makes use of all available information and outperforms alternative approaches such as listwise deletion, pairwise deletion, or data imputation in SEM contexts^43^. Missingness resulted from adolescents being allowed to answer *I don’t know* or *Prefer not to say* or skip any question in the questionnaire. Across variables, 1.4–13.8% of data were missing — up to 2.8% for body esteem, 3.3% for self-objectification, 13.8% for appearance activity, and 12.7% for resilience. The variances of latent variables were fixed at 1. Model fit evaluation relied on conventional thresholds (TLI ≥ 0.90, RMSEA ≤ 0.08, SRMR ≤ 0.10)^42^. All variables were modeled as reflective latent. Latent interaction terms were estimated using the product-indicator method^44^, in which interactions were formed by multiplying all possible pairs of observed indicators of the respective latent variables. Gender differences in the main relationships between appearance activity, resilience to media ideals and to negative appearance feedback, body esteem, and self-objectification were examined by comparing a model constraining these paths to be equal across groups with an unconstrained model. Gender differences in moderation effects were evaluated only via effect sizes, as the SEM models’ fit with latent interactions tends to be unstable^44^ and thus not meaningful to compare.

In the first model, which estimated main effects, appearance activity on social media and resilience to media ideals and to negative appearance feedback were entered as predictors, with body esteem and self-objectification as dependent variables. In the second model, which examined moderation effects, appearance activity was entered as a predictor, and resilience to media ideals and to negative appearance feedback were entered as both predictors and moderators. All analysis scripts and data used in this study are available at OSF (https://osf.io/69ncp/overview?view_only=0169d7a7034a443f952fd0b5d6f15a43).

### Deviations from preregistration

Although the preregistered sample included 927 adolescents aged 15–19 (*M* = 16.97, *SD* = 1.19; 59% girls, 38% boys, 4% other genders), the final sample consisted of 885 adolescents aged 15–19 (*M* = 16.99, *SD* = 1.19; 61% girls, 39% boys). Because the multi-group models compared girls and boys, 34 adolescents who identified as another gender were excluded, and an additional eight adolescents were omitted due to missing gender information. In addition to the preregistered research questions, we added an additional one regarding gender differences in the potential moderation roles of resilience to media ideals and to negative appearance feedback.

When testing measurement invariance, we allowed one cross-loading and residual covariances between several items. For the body esteem scale, a residual covariance between two pairs of items (i.e., *There are lots of things I’d change about my looks if I could* with *I wish I looked better; I feel ashamed of how I look* with *I worry about the way I look.*) was freely estimated. Their residual covariances were likely due to the proximity of reverse-coded items in the scale and their overlapping content. One modification involved a cross-loading in the self-objectification scale: the item belonging to the "thinking of body as representing self" dimension (i.e., *Looking attractive to others is more important to me than being happy with who I am inside*) also loaded on the "observer’s perspective on body" dimension, possibly due to item proximity and overlapping content. For the appearance activity scale, residual covariances between two pairs of items (i.e., W*hen on social media my friends post, comment on, share or like content about getting or staying fit and/or muscular* with *When on social media my friends post, comment on, share or like content about what and when to eat; When on social media I post, comment on, share or like content about what and when to eat* with *When on social media my friends post, comment on, share or like content about what they would like their bodies to look like*) were freely estimated.

Contrary to preregistration, appearance activity was modeled as a higher-order factor with two lower-order factors: activities adolescents engage in themselves and activities they observe their friends engaging in on social media. This decision was based on both modification indices within measurement invariance testing and theoretical justification for distinguishing between one’s own activities and those of friends. Further, we did not test measurement invariance for the resilience to media ideals scale because the two-indicator model was not identified. We also aimed initially to estimate dynamic cutoffs for both measurement invariance testing and the main SEM model; however, dynamic cutoffs are not yet available for complex SEM models. Dynamic cutoffs were then used only for invariance testing, and traditional cutoffs were used to evaluate the main SEM model.

## Results

Zero-order correlations among the used variables are presented in Table 1. Adolescent boys reported slightly higher body esteem and lower self-objectification than girls. They also reported lower resilience to body ideals in the media and to negative appearance feedback. More frequent engagement in appearance activity on social media was correlated with heightened self-objectification and reduced body esteem. While higher resilience to negative appearance feedback was linked to slightly higher body esteem and lower self-objectification, resilience to media ideals showed the opposite pattern, correlating with lower body esteem and higher self-objectification.

**Table 1:** Zero-order correlations among variables used in analyses.

	(1)	(2)	(3)	(4)	(5)	(6)	(7)
(1) Gender	1						
(2) Age	0.15**	1					
(3) Body esteem	0.25**	0.08*	1				
(4) Self-objectification	− 0.24**	− 0.12**	− 0.49**	1			
(5) Appearance activity	− 0.02	− 0.03	− 0.21**	0.26**	1		
(6) Resilience – ideals	− 0.25**	− 0.14**	− 0.23**	0.29**	0.19**	1	
(7) Resilience – feedback	− 0.18**	− 0.03	0.14**	− 0.13**	− 0.08*	0.25**	1

**p < 0.010; *p < 0.050. Gender was coded as 1 = *girls*, 2 = *boys*.

### Measurement invariance

The results of measurement invariance testing between girls and boys are presented in Table 2. All scales mostly achieved metric invariance according to both traditional and dynamic measurement invariance cutoffs, with a few exceptions. RMSEA values for appearance activity and resilience to negative appearance feedback were worse than expected, and body esteem also showed slightly poorer RMSEA than ideal based on traditional cutoffs. Metric invariance, indicated by changes in fit indices from the configural model, was fully achieved for self-objectification, appearance activity, and resilience to negative appearance feedback according to both types of indices, while the body esteem scale slightly exceeded the dynamic cutoff for CFI and RMSEA.

**Table 2:** Results of measurement invariance between girls and boys.

Construct	Invariance	df	BIC	*X*^2^	*p*	TLI	CFI	SRMR	RMSEA	90% CI low	90% CI high
BE	Configural	50	19,237.58	212.13	< 0.001	0.944	0.961	0.036	0.094	0.081	0.107
	Metric	58	19,205.47	232.72	< 0.001	0.948	0.958	0.053	0.090	0.078	0.102
	Scalar	66	19,207.47	284.82	< 0.001	0.943	0.948	0.058	0.094	0.083	0.105
SO	Configural	150	34,834.12	420.15	< 0.001	0.912	0.927	0.052	0.069	0.062	0.077
	Metric	163	34,765.91	440.18	< 0.001	0.917	0.925	0.056	0.067	0.060	0.075
	Scalar	175	34,724.86	478.56	< 0.001	0.916	0.916	0.060	0.068	0.061	0.075
AA	Configural	12	18,774.78	57.46	< 0.001	0.900	0.960	0.032	0.123	0.092	0.156
	Metric	16	18,757.17	66.63	< 0.001	0.919	0.957	0.040	0.111	0.085	0.139
	Scalar	20	18,734.04	74.14	< 0.001	0.935	0.957	0.041	0.099	0.076	0.123
RES	Configural	4	13,192.60	19.77	< 0.001	0.929	0.976	0.032	0.122	0.071	0.180
	Metric	7	13,174.90	24.23	< 0.001	0.961	0.977	0.035	0.091	0.053	0.132
	Scalar	10	13,202.38	64.65	< 0.001	0.919	0.932	0.068	0.131	0.101	0.162

BE Body esteem, SO Self-objectification, AA Appearance activity on social media, RES Resilience to negative appearance feedback.

Overall, metric measurement invariance was considered sufficient for the purposes of this study, though the limitations of some coefficients not fully supporting it should be acknowledged. Establishing metric invariance indicates that factor loadings are equivalent across groups, allowing for meaningful comparisons of latent (co)variances, covariances, and structural paths between groups.

According to dynamic cutoffs, scalar invariance was achieved only for body esteem and self-objectification (except for SRMR values). Resilience to negative appearance feedback and appearance activity showed significantly different intercepts between groups and thus did not achieve scalar invariance, limiting the possibility of comparing latent means between girls and boys. Although scalar invariance was not required for this study, these results may inform future research.

### Roles of appearance activity on social media and resilience in body esteem and self-objectification

The model in which social media appearance activity and resilience to body ideals in media and to negative appearance feedback predicted body esteem and self-objectification fit reasonably well, although TLI and CFI values were slightly lower.: χ2 (1119) = 2494.813, RMSEA = 0.057, 95% CI [0.054; 0.060], CFI = 0.890, TLI = 0.883, SRMR = 0.079. The results are presented in Table 3.

**Table 3:** The results for the associations between social media appearance activity, resilience to body ideals in media, resilience to negative appearance feedback, self-objectification, and body esteem.

		*B*	*SE*	β	95% CI		*p*
					Lower	Upper	
*Girls*	Self-objectification						
	Appearance activity	0.20	0.07	**0.21**	0.07	0.35	**0.005**
	Resilience – feedback	− 0.41	0.08	− 0**.36**	− 0.50	− 0.23	** < 0.001**
	Resilience – ideals	0.19	0.04	**0.34**	0.21	0.47	** < 0.001**
	Body esteem						
	Appearance activity	− 0.19	0.06	− 0**.19**	− 0.30	− 0.07	**0.002**
	Resilience – feedback	0.32	0.07	**0.27**	0.16	0.38	** < 0.001**
	Resilience – ideals	− 0.13	0.03	− 0**.22**	− 0.32	− 0.12	** < 0.001**
*Boys*	Self-objectification						
	Appearance activity	0.38	0.10	**0.34**	0.19	0.50	** < 0.001**
	Resilience – feedback	− 0.18	0.09	− 0**.16**	− 0.31	− 0.01	**0.036**
	Resilience – ideals	0.18	0.07	**0.16**	0.04	0.28	**0.009**
	Body esteem						
	Appearance activity	− 0.05	0.08	− 0.04	− 0.19	0.11	0.580
	Resilience – feedback	0.21	0.08	**0.21**	0.06	0.35	**0.008**
	Resilience – ideals	− 0.21	0.07	− 0**.20**	− 0.32	− 0.08	**0.001**

Statistically significant values at *p* < 0.050 are in bold.

The results indicated gender differences in the examined relationships (constrained model, χ2 (1125) = 2727.9, unconstrained model, χ2 (1119) = 2714.5, *p* = 0.041). More frequent social media appearance activity was associated with lower body esteem in girls, but not in boys, who showed no significant association. Other associations, however, were similar between girls and boys. More frequent appearance activity was linked to higher self-objectification. Higher resilience to negative appearance feedback was related to higher body esteem and lower self-objectification. Resilience to body ideals in media showed an unexpected relationship: higher resilience was associated with lower body esteem and greater self-objectification.

### Moderation by resilience between appearance activity on social media, self-objectification, and body esteem

**Fig. 1:**
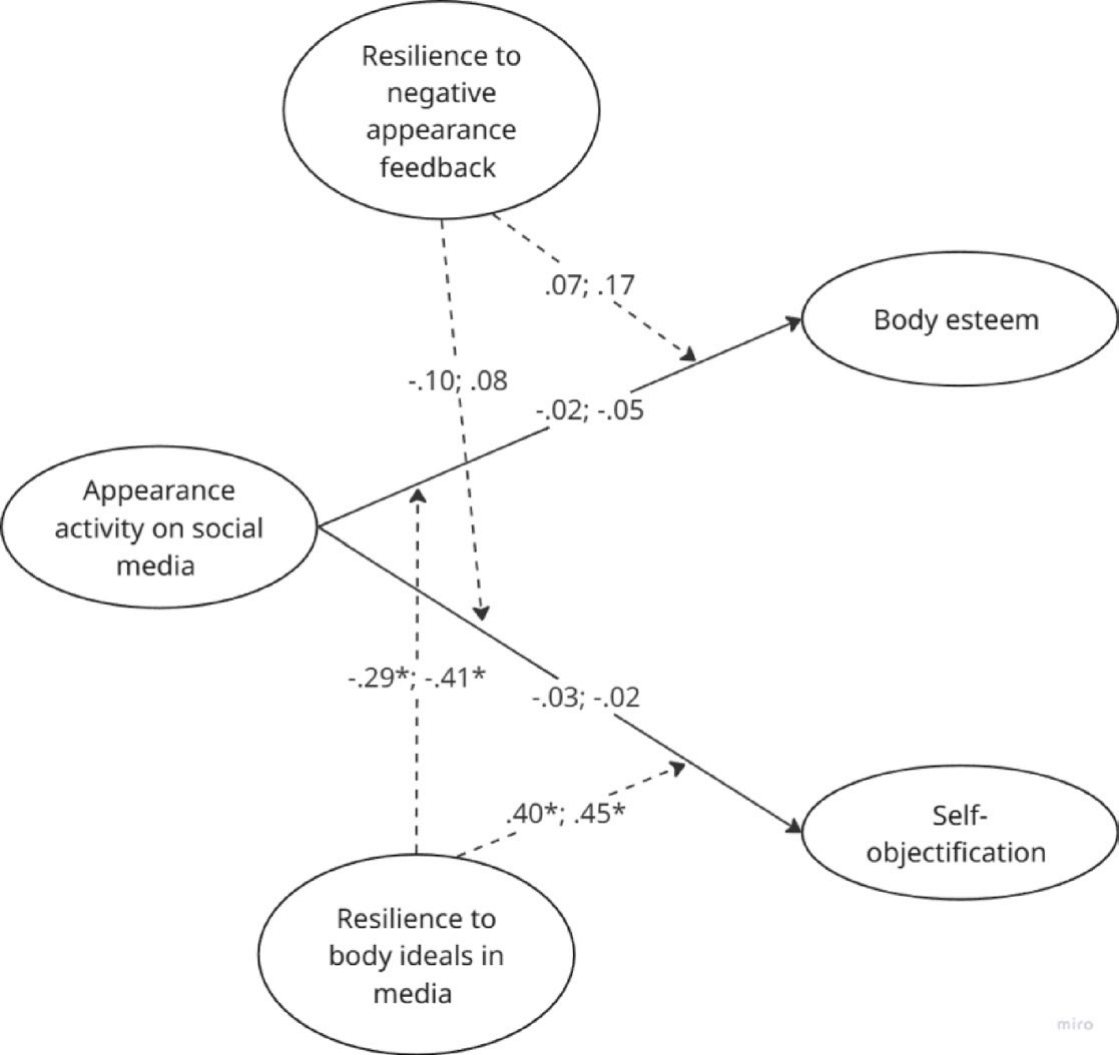
Moderation by resilience to media ideals and to negative appearance feedback in the links between social media appearance activity, body esteem, and self-objectification.

Resilience to negative appearance feedback did not significantly moderate the associations between social media appearance activity and body esteem (*girls*: *B* = 0.07, *p* = 0.354; *boys: B* = 0.17, *p* = 0.060) or self-objectification (*girls: B* = − 0.10, *p* = 0.299; *boys: B* = 0.08, *p* = 0.487). In contrast, resilience to body ideals in media did significantly moderate these associations for both body esteem (*girls: B* = − 0.29, *p* = 0.008; *boys: B* = − 0.41, *p* = 0.002) and self-objectification (*girls: B* = 0.40, *p* = 0.008; *boys: B* = 0.45, *p* = 0.005), but in an unexpected direction. Girls and boys showed no differences in these moderations. The results for the moderations and main effects are presented in Fig. 1.

**Fig. 2:**
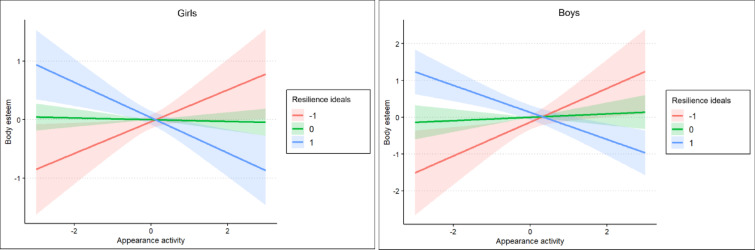
Moderation of the link between social media appearance activity and body esteem by resilience to media ideals.

**Fig. 3:**
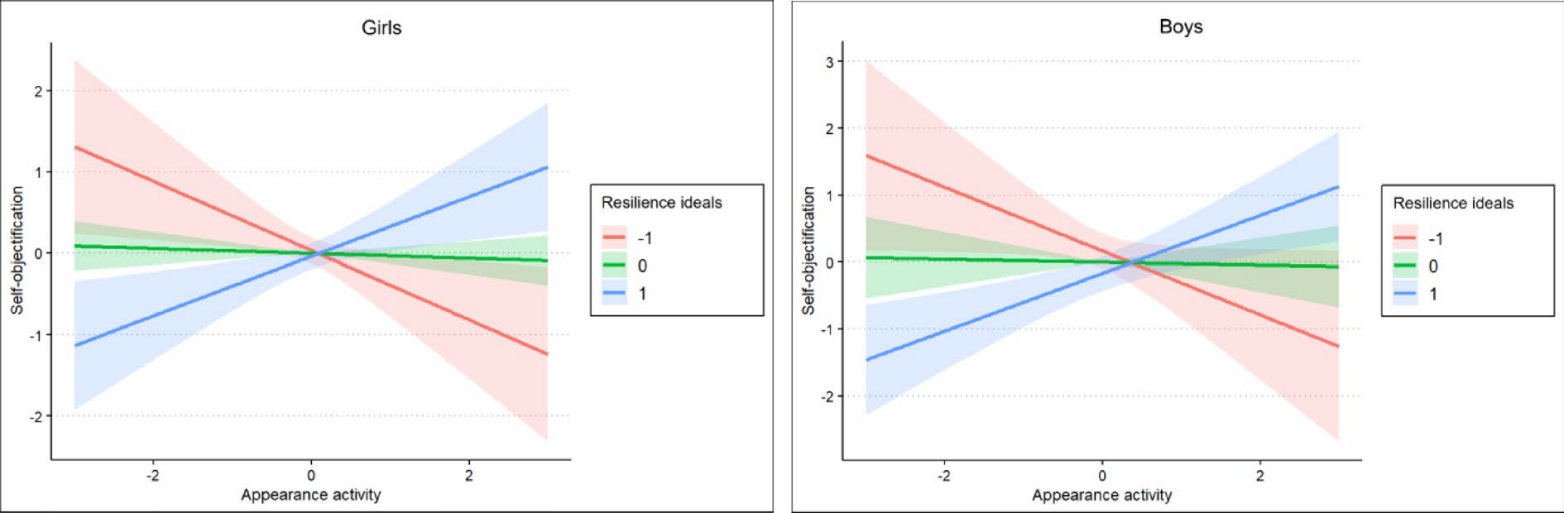
Moderation of the link between social media appearance activity and self-objectification by resilience to media ideals.

Simple slope analyses clarified these effects. As shown in Table 4 and Figs. 2 and 3, higher appearance activity predicted higher body esteem and lower self-objectification at the lowest levels of resilience to media ideals, but lower body esteem and higher self-objectification at the highest levels of resilience. At average levels of resilience to media ideals, appearance activity was not significantly associated with either body esteem or self-objectification. These patterns were observed in both girls and boys.

**Table 4:** Simple slopes of social media appearance activity predicting body esteem and self-objectification at low, average, and high levels of resilience to media ideals.

	Girls			Boys		
	B	SE	*p*	B	SE	*p*
Body esteem						
− 1 SD	**0.46**	0.19	**0.017**	**0.27**	0.13	**0.036**
Mean	0.05	0.08	0.560	− 0.01	0.04	0.703
+ 1 SD	− **0.37**	0.10	** < 0.001**	− **0.30**	0.10	**0.002**
Self-objectification						
− 1 SD	− **0.48**	0.24	**0.046**	− **0.43**	0.18	**0.018**
Mean	− 0.02	0.10	0.832	− 0.03	0.05	0.566
+ 1 SD	**0.43**	0.13	**0.001**	**0.37**	0.13	**0.005**

Statistically significant values at *p* < 0.050 are in bold.

We note that two sensitivity analyses were conducted to examine the robustness of the moderation effects. The results suggested that resilience to negative appearance feedback may also play a role (when tested in separate models); however, its effects were likely not substantial enough and robust, as their statistical significance depended on the method of scaling the latent variables (see Appendix B for details).

## Discussion

This study explored whether resilience to negative appearance feedback and to body ideals in media play protective roles in adolescents’ body image and mitigate the associations between social media appearance activity and heightened self-objectification and decreased body esteem. Although this study mainly focused on the moderation by resilience, it is worth noting that the more girls engaged in appearance activity on social media, the more they reported lower body esteem and higher self-objectification. This is consistent with the notion that social media can lead adolescents to overemphasize their appearance and feel less satisfied with their bodies due to the promotion of unattainable ideals^28^. Boys, however, showed only heightened self-objectification with more frequent engagement in appearance activity, with no significant link to body esteem. As boys tend to engage less seriously with appearance-related content^29^ and as they are more inclined toward body ideals for functional rather than appearance reasons (e.g., physical strength)^45^, they may be less susceptible to reduced body esteem in relation to social media appearance activity.

Unexpected results emerged for the associations between resilience and body image variables. While both resilience to negative appearance feedback and resilience to media ideals were considered protective and expected to be linked with higher body esteem and lower self-objectification^7,30^, only resilience to negative appearance feedback showed this. Resilience to media ideals had quite the opposite association: adolescent girls and boys who reported higher resilience to media ideals also reported lower body esteem and heightened self-objectification. Bearing in mind the preliminary exploratory nature of these results, this points to the potentially intricate role of resilience to media ideals when adolescents engage with visual social media. As further discussed below, these relationships may also reflect the reverse direction, where body image concerns could stimulate the development of resilience against idealized bodies^30,31^.

While we expected resilience to shield adolescents from the idealization and emphasis on appearance encouraged by visual social media, the results did not show this. Resilience to negative appearance feedback did not moderate these associations, which is inconsistent with the expectation that such resilience, as a crucial component of positive body image^7^, should buffer the associations between social media appearance activity and body image concerns (i.e., reduced body esteem and heightened self-objectification). Resilience to media ideals did moderate these relationships, but showed a different picture than anticipated because it strengthened the association between more frequent appearance activity and body image concerns. Among both girls and boys with higher-than-average resilience, more frequent engagement in appearance activity on social media was associated with heightened self-objectification and decreased body esteem. While these relationships were not significant among adolescents with average resilience scores, appearance activity was linked to higher body esteem and lower self-objectification among those reporting lower-than-average resilience. These results were surprising, given that resilience should help adolescents cope with body image threats on social media^24^ and mitigate the links between appearance activity and body image concerns.

We believe it is important to note that, given the exploratory and cross-sectional study, these results should not be interpreted as conclusive evidence that resilience to media ideals makes adolescents vulnerable to visual social media engagement and puts their body image at risk. This caution is further warranted by the sensitivity analyses, which indicated that although the moderation by resilience to media ideals remained statistically significant, the simple slopes were not when an alternative method of latent variable scaling was applied. The simple slope analysis may have been underpowered, as it requires dividing the sample into thirds based on resilience values. Still, the results preliminarily hint that resilience to media ideals may play an intricate role. We invite further research to corroborate these results across different research designs and diverse samples.

A few reflections can be drawn from these results. Although we assumed that resilience would be associated with better coping with idealized bodies and negative comments on social media, the unexpected results for resilience to media ideals may also reflect a reverse link, where resilience develops as a result of engaging with these ideals and experiencing body image concerns. A similar mechanism has been observed, for example, among women struggling with eating disorders, who disclosed becoming more resilient from their adverse body-related experience^46^. This might explain why higher resilience to media ideals was linked with lower body esteem and heightened self-objectification as such (i.e., main effect) or in relation to more frequent social media appearance activity (i.e., a moderating effect). This becomes particularly true if the association from body image concerns to resilience was stronger than vice versa, which emphasizes the need to disentangle the potential bidirectional relationships between resilience, appearance-related social media engagement, and body image concerns in future research.

Another reflection relates to the measurement of resilience. Resilience was measured by asking adolescents about their actual responses to attractive ideals or negative appearance feedback, but also about their potential reactions (i.e., how they would or try to respond, unlike adult resilience and coping scales, which typically focus on actual responses to such threats; for an overview, see^47^). The measurement used in this study was not limited to having unpleasant body image-related experience, and such resilience has been identified as an important component of positive body image during adolescence^7^. However, it may mix adolescents who actually employ protective strategies with those who are aware of them but find them difficult to use. Likely, the relationship between engagement in social media appearance activity and body image concerns may be buffered by adolescents’ actual ability to distract themselves from negative messages and avoid placing importance on them. As a thought, resilience, conceptualized as including potential reactions to media ideals, may also unintentionally capture a greater awareness of the idealization on social media, which could paradoxically reinforce body image concerns^48^. This can be particularly pronounced when adolescents struggle to employ protective strategies against idealized content. On the other hand, protective strategies against idealized body images and negative appearance-related feedback may be partly automatic and habitual, as has been observed in social media behaviors^49^. Consequently, adolescents might engage in these strategies without being fully aware of doing so and therefore may not report them, which could also explain the absence of a protective effect of resilience.

Besides the unexpected moderation by resilience to media ideals, resilience to negative appearance feedback did not appear as a moderator. This is somewhat counterintuitive, because seeking appearance feedback is one of the main motivations for social media use among adolescents^50^, and the platforms facilitate this feedback through likes and comments on selfies and other images, both in feeds and via direct messages. Resilience should therefore buffer against potential resultant body image concerns, particularly given that, unlike resilience to media ideals, this form of resilience was associated with higher body esteem and lower self-objectification in the present study, and with improved body image in the previous work^7^. One possible explanation is that resilience to negative appearance feedback may be less salient when adolescents engage with appearance-related feedback (e.g., comments, likes, reposts) on idealized social media content as opposed to feedback in group chats or offline contexts. Idealized content typically depicts attractive appearance ideals, and reactions to such images may be more strongly influenced by resilience to these ideals. Somewhat consistent with this reasoning is the notion that idealized images may exert stronger effects on body image than the accompanying likes and commentary on social media^51^.

It should be noted, however, that moderation by resilience to negative appearance feedback cannot be completely ruled out, as alternative models in which this moderator was entered alone (without resilience to media ideals) showed similar patterns to those for resilience to media ideals (see Appendix B). Although follow-up research is encouraged to explore the interplay between these types of resilience more thoroughly, the present study showed that resilience to negative appearance feedback did not substantially add to the explanatory power of resilience to media ideals, which may be more salient in the context of social media appearance activity that dominantly portrays appearance ideals.

We did not find gender differences in the moderation of resilience to media ideals or to negative appearance feedback. Both girls and boys showed similar patterns: resilience to media ideals appeared to strengthen the link between more frequent appearance activity and body image concerns, while lower resilience was linked with fewer concerns. Resilience to negative appearance feedback did not moderate these associations in either group. This may suggest that girls and boys experience resilience on social media similarly, echoing previous research showing no gender differences in social media effects on body image^28^. Although boys have been found to report more protective strategies in qualitative studies^26^, they indicated lower resilience than girls in our study. Although this may partly reflect gender norms, where boys are expected to care less about appearance, it also points to potential differences in how adolescents navigate idealization and appearance-related feedback on social media.

### Limitations and implications

We should acknowledge the limitations of the measurement scales, which did not show ideal model fit in the measurement invariance tests and required adjustments by allowing cross-loading and residual covariances. This may limit the generalizability of the results to some extent and should be taken into account. Another point to consider is that resilience to body ideals was assessed broadly across media and did not capture features specific to appearance-related social media content, such as the endorsement of idealized bodies through “likes” and comments or their particular framing, which may further exacerbate (e.g., when idealized bodies are presented within body-positive posts,^52^) or mitigate body image concerns (e.g., challenging “reality check” comments^53^). Incorporating these platform-specific characteristics into the assessment of resilience may reveal stronger protective effects in the context of engagement with appearance-focused social media content. Furthermore, social media appearance activity, resilience, and body image variables may have been influenced by individual and contextual factors that were not accounted for. For example, body dissatisfaction may be shaped by parental communication or experiences of weight-based teasing^54^, whereas self-objectification may be affected by receiving appearance-based commentary or one’s attractiveness^55^. Parental and peer factors—such as growing up in a more or less body-accepting environment—likewise shape resilience in the body image domain and engagement in appearance-related social media practices^23,24^. Incorporating these factors could provide clearer insight into the links between these body image variables, resilience, and social media appearance activity.

Nonetheless, this study brings preliminary results for resilience to attractive media ideals and to negative appearance feedback as salient components of positive body image^7^, suggesting that resilience to attractive ideals may not buffer late adolescent girls and boys from the body image concerns associated with engagement in social media appearance activity. Although these results could hint that resilience may increase vulnerability to these concerns, given the absence of other research, the need for further studies cannot be stressed enough. Follow-up studies could implement longitudinal and experimental methodologies to investigate whether and how resilience shapes reactions to idealized social media content and their effects on body image. This is especially needed given that our cross-sectional design did not allow us to infer causality or to examine the likely bidirectional relationships between appearance-related activity, resilience, and body image concerns. Further, a qualitative inquiry into the conditions under which resilience to media ideals may expose adolescents to body image concerns also seems warranted. These studies could also account for the complexity in visual social media and body image. These could include engagement with body-positive social media, which encourages body acceptance and may improve body image^56^, as well as positive body image outcomes that extend beyond body esteem, such as the appreciation of one’s own and others’ bodies^7^ and embracing body functionality and body care^57^. Although the protective role of resilience was not shown in our study on social media appearance activity, resilience could enhance the positive body image gained from engagement with body-positive content.

Prevention efforts should not be discouraged from fostering resilience and protective strategies against body image–related threats, because these play a protective role in adolescents’ body image^7,58^. Further research is needed to clarify how resilience functions in the context of visual social media. Given that body image concerns can spill over into worsened health outcomes, such as depressive and anxiety symptoms^11,12^ and eating disturbances^13,14^, understanding when resilience alleviates, or, potentially, exacerbates body image concerns should inform the development of strategies to promote well-being during adolescence.

## Conclusions

As adolescents regularly interact with unrealistically idealized bodies on social media^1^, resilience to these pressures may protect their body image^7^, although research on this important issue has been lacking. The present study explored this question and found that, while resilience to negative appearance feedback did not moderate the relationship between social media appearance activity, self-objectification, and body esteem, resilience to media ideals did, but in an unexpected direction. Adolescents with the highest resilience showed more pronounced body image concerns in relation to their engagement with social media appearance activity, whereas adolescents with the lowest resilience showed the opposite: more frequent engagement was associated with higher body esteem and lower self-objectification. These results suggest that resilience may not necessarily be protective for adolescents’ body image when engaging with appearance-focused content on social media. They highlight the need for follow-up studies to examine this important aspect of adolescent body image and to establish the conditions under which resilience shields adolescents in the context of visual social media.

## Data Availability

The dataset analyzed in this study is available at OSF (https://osf.io/69ncp/overview?view_only=0169d7a7034a443f952fd0b5d6f15a43).

## Appendix A

The standard cut-offs were: good/acceptable fit criteria for configural model, RMSEA ≤ 0.06/0.08, SRMR ≤ 0.08, TLI/CFI ≥ 0.95/0.90^40^; maximum differences between configural and metric models defined as ΔCFI ≤ 0.01, ΔRMSEA ≤ 0.015, and ΔSRMR ≤ 0.030; maximum differences between the scalar to metric model (Table 5), ΔCFI ≤ 0.01, ΔRMSEA ≤ 0.015, and ΔSRMR ≤ 0.015^41^.

**Table 5:** Dynamic cutoffs for measurement invariance testing.

	Change statistics	Invariance	
		Metric	Scalar
Body esteem	ΔRMSEA	0.003	0.007
	ΔSRMR	0.025	0.005
	ΔCFI	− 0.002	− 0.003
Self-objectification	ΔRMSEA	0.002	0.002
	ΔSRMR	0.011	0.002
	ΔCFI	− 0.004	− 0.004
Appearance activity	ΔRMSEA	0.018	0.017
	ΔSRMR	0.014	0.005
	ΔCFI	− 0.004	− 0.005
Resilience feedback	ΔRMSEA	0.037	0.03
	ΔSRMR	0.026	0.01
	ΔCFI	− 0.007	− 0.005

## Appendix B

### Sensitivity analyses

Here, we describe the results of two sensitivity analyses: (a) a model in which the variances of latent factors were not fixed to 1 (and were indicated by fixing the first factor loading to 1 instead), and (b) models in which latent interaction terms were tested separately for each moderator rather than together in a single model.

### Model with latent factors scaled by a factor loading (instead of fixing variances to 1)

Same as the main model reported in the paper, this model showed significant moderation effects for resilience to body ideals in media in the links between social media appearance activity, self-objectification (*girls: B* = 0.02, *p* = 0.009; *boys: B* = 0.03, *p* = 0.018), and body esteem (*girls: B* = − 0.03, *p* = 0.007.; *boys: B* = − 0.05, *p* = 0.015). However, the simple slopes were not statistically significant, as detailed below.

We favored the model with standardized latent variances because it allows comparison between individual predictors, placing them on the same scale. However, the differences observed in the simple slopes analyses suggest that these effects may not be entirely robust (Table 6).

**Table 6:** Alternative model: simple slopes of social media appearance activity predicting body esteem and self-objectification at low, average, and high levels of resilience to media ideals.

	Girls			Boys		
	B	SE	*p*	B	SE	*p*
Body esteem						
− 1 SD	0.02	0.03	0.468	0.11	0.10	0.258
Mean	− 0.01	0.03	0.719	0.06	0.08	0.466
+ 1 SD	− 0.04	0.02	0.067	0.00	0.06	0.942
Self-objectification						
− 1 SD	− 0.04	0.03	0.148	− 0.06	0.06	0.350
Mean	− 0.01	0.02	0.524	− 0.03	0.05	0.604
+ 1 SD	0.01	0.02	0.451	0.01	0.04	0.900

### Separate models for each moderation variable: resilience to negative appearance feedback and resilience to body ideals in media

When examining moderation by resilience to media ideals and resilience to negative appearance feedback in separate models, resilience to negative appearance feedback significantly moderated the association between social media appearance activity and self-objectification (*girls: B* = 0.16, *p* = 0.003; *boys: B* = 0.28, *p* = 0.014). Moderation in the link between appearance activity and body esteem was also significant for girls (*B* = − 0.12, *p* = 0.009). All effects were in the same direction as in the main analysis. However, because these moderation effects were not significant in the main model, we reason that their explanatory power was limited compared to resilience to media ideals. Still, it is possible that the nonsignificance in the main model was caused by other factors, such as higher model complexity and slightly larger standard errors (Table 7).

**Table 7:** Alternative model: simple slopes of social media appearance activity predicting body esteem and self-objectification at low, average, and high levels of resilience to negative appearance feedback.

	Girls			Boys		
	B	SE	*P*	B	SE	*p*
Body esteem						
− 1 SD	0.00	0.07	0.972	− 0.12	0.12	0.301
Mean	− **0.12**	0.04	**0.002**	− **0.13**	0.05	**0.014**
+ 1 SD	− **0.24**	0.05	** < 0.001**	− **0.15**	0.07	**0.025**
Self-objectification						
− 1 SD	− 0.05	0.08	0.568	− 0.10	0.18	0.574
Mean	**0.11**	0.05	**0.021**	**0.18**	0.09	**0.038**
+ 1 SD	**0.28**	0.06	** < .001**	**0.45**	0.09	** < .001**

Statistically significant values at *p* < 0.050 are in bold.

The moderation model with resilience to body ideals in media (without the other moderator) showed patterns similar to the main model, with significant moderation effects for body esteem (*girls: B* =  − 0.29, *p* < 0.001; *boys: B* = − 0.40, *p* = 0.002) and self-objectification (*girls: B* = 0.37, *p* < 0.001; *boys: B* = 0.62, *p* = 0.001). Simple slopes results are reported below (Table 8).

**Table 8:** Alternative model: simple slopes of social media appearance activity predicting body esteem and self-objectification at low, average, and high levels of resilience to body ideals in media.

	*Girls*			*Boys*		
	*B*	*SE*	*p*	*B*	*SE*	*p*
Body esteem						
− 1 SD	**0.27**	0.13	**0.036**	**0.46**	0.19	**0.017**
Mean	− 0.01	0.04	0.702	0.05	0.08	0.560
+ 1 SD	− **0.30**	0.10	**0.002**	− **0.37**	0.10	** < 0.001**
Self-objectification						
− 1 SD	− **0.46**	0.17	**0.006**	− **0.48**	0.24	**0.046**
Mean	− 0.09	0.08	0.265	− 0.02	0.10	0.832
+ 1 SD	**0.28**	0.08	**0.001**	**0.43**	0.13	**0.001**

Statistically significant values at *p* < 0.05 are in bold.

### Notes on analyses

It should be noted that the model with latent interaction effects produced a non-positive definite matrix warning of latent variable correlations. Upon inspection, no negative variances or other problematic parameters were observed, and the matrix determinant was nonzero (*D* = 0.007), indicating there should be no linear dependencies. The warning was likely due to high correlations between the predictor variables (social media appearance activity, resilience to media ideals, and resilience to negative appearance feedback) and their latent interaction terms. Because the interaction terms were computed from these predictors’ indicators, such high correlations are tolerable, particularly given the small number of indicators in the resilience scales, which likely inflated these correlations. Therefore, we considered the model reliable despite the warning.
